# Ultrafast photocurrent detection contradicts optical detection conclusions: Exciton diffusion contributes little to carbon nanotube device efficiency

**DOI:** 10.1126/sciadv.aec3186

**Published:** 2026-07-01

**Authors:** Zachary M. Faitz, Christopher J. Blackwell, Dasol Im, Abitha Dhavamani, Xingyu Shen, Michael S. Arnold, Martin T. Zanni

**Affiliations:** ^1^ Department of Chemistry, University of Wisconsin–Madison, Madison, WI 53706, USA.; ^2^ Department of Materials Science and Engineering, University of Wisconsin–Madison, Madison, WI 53706, USA.

## Abstract

Many conclusions about energy conversion in next-generation photovoltaic devices are gleaned indirectly from optical measurements of exciton dynamics, not directly from photocurrent itself. This method is problematic because optical measurements report on all excitons, not just productive ones. Using a new ultrafast photocurrent spectrometer, we compare exciton dynamics of semiconducting carbon nanotubes measured in films to those measured in devices using photoabsorption- and photocurrent-detected transient and two-dimensional spectroscopies. We find that photoabsorption detection greatly overestimates the importance of long-lived excitons for photovoltaic device performance. Excitons diffuse across nanotubes for picoseconds, but we find that the photocurrent is mostly created by excitons that diffuse little before dissociating at the electron transfer interface within 30 femtoseconds of being created. Thus, scientific conclusions reached from optical-only studies bear little importance to the performance of these devices, calling into question the processes thought critical for efficient photoconversion. This study points to the necessity for directly measuring photocurrent-generating exciton dynamics, not surmising them from optical spectroscopy alone.

## Teaser

Ultrafast photocurrent spectroscopy reveals information on solar cells that were previously studied with optical spectroscopy.

## INTRODUCTION

Many next-generation solar cells generate photocurrent by harvesting excitons (*1*–*5*). In this photovoltaic device design, photons generate electron-hole pairs (excitons), which are separated into free carriers at the interface of electron and hole transfer materials. The charges flow through their respective domains to the electrodes, creating current. To generate current, excitons must reach an interface before recombining. Thus, exciton dynamics are thought to be intricately linked to device efficiency in this class of next-generation photovoltaics (*6*–*9*).

The most common approach for characterizing exciton dynamics is using ultrafast transient absorption (or pump-probe) spectroscopy on a thin film (*9*–*13*). This approach has two assumptions: The properties of the photoactive material are the same in a film as in a photovoltaic device, and transient absorption captures the dynamics of excitons responsible for photocurrent. Regarding the former, the morphology of a thin film may differ in a photovoltaic device, film studies usually lack an electron-accepting material, and there are no electric fields in a film. Regarding the latter, absorption detection measures all excitons within the probe’s bandwidth, regardless of the exciton’s fate, providing an average of all exciton dynamics. Even the best next-generation photovoltaics do not create current from every photon absorbed, so discerning the productive exciton pathways relevant for device performance from transient absorption is not straightforward.

Ideally, one would measure exciton dynamics within the photovoltaic device itself by implementing both photocurrent and absorption detection. Photocurrent detection does not report on recombined or trapped excitons, only measuring excitons that ultimately generate current (*14*). Using both absorption and photocurrent detection would characterize current-generating and non–current-generating pathways, thereby revealing excitons that contribute to device efficiency and those that do not.

Photocurrent detection is commonly used to evaluate photovoltaics, but it is rarely applied in ultrafast pump-probe and two-dimensional (2D) experiments because of an “incoherent” background (*15*–*17*). This background is created by exciton-exciton, exciton-charge, and/or charge-charge interactions that occur after the probe pulse and can be orders of magnitude larger than the desired pump-probe signals (*18*–*22*). Recently, two methods for removing the incoherent signals have been reported (*23*, *24*). Here, we use one of these techniques, a new polarization scheme that gives background-free, photocurrent-detected spectra in isotropically ordered samples (*23*).

In this work, we use films and photovoltaic devices made from single-walled, semiconducting carbon nanotubes (CNTs) acting as the light-absorbing and exciton transport material (*25*). CNTs are promising materials for photovoltaics because they are extremely strong absorbers, have an easily tunable bandgap based on their diameter [referred to by their chiral index (*n*,*m*)], are chemically stable, and solution-processable (*3*, *26*, *27*). Excitons are highly stable in CNTs because their one-dimensional nature reduces dielectric screening. In addition, their wire-like geometry and fast exciton diffusivity allow for rapid exciton transport (>100 nm along their length). Moreover, their narrow absorption linewidths (~40 nm) make them excellent materials for spectroscopic study because exciton and charge transfer can be monitored between different bandgap CNTs. We know from transient absorption spectroscopy that excitons diffuse and transfer in these films for several picoseconds, which has guided CNT photovoltaic design (*28*). Here, we report that transient absorption spectroscopy vastly overestimates the importance of intertube exciton hopping and diffusion for photovoltaic device performance because the photocurrent is created almost entirely by excitons, requiring little to no diffusion. These realizations are leading us to reevaluate the photophysics that dictate CNT photovoltaic device function.

## RESULTS

**Fig. 1.**
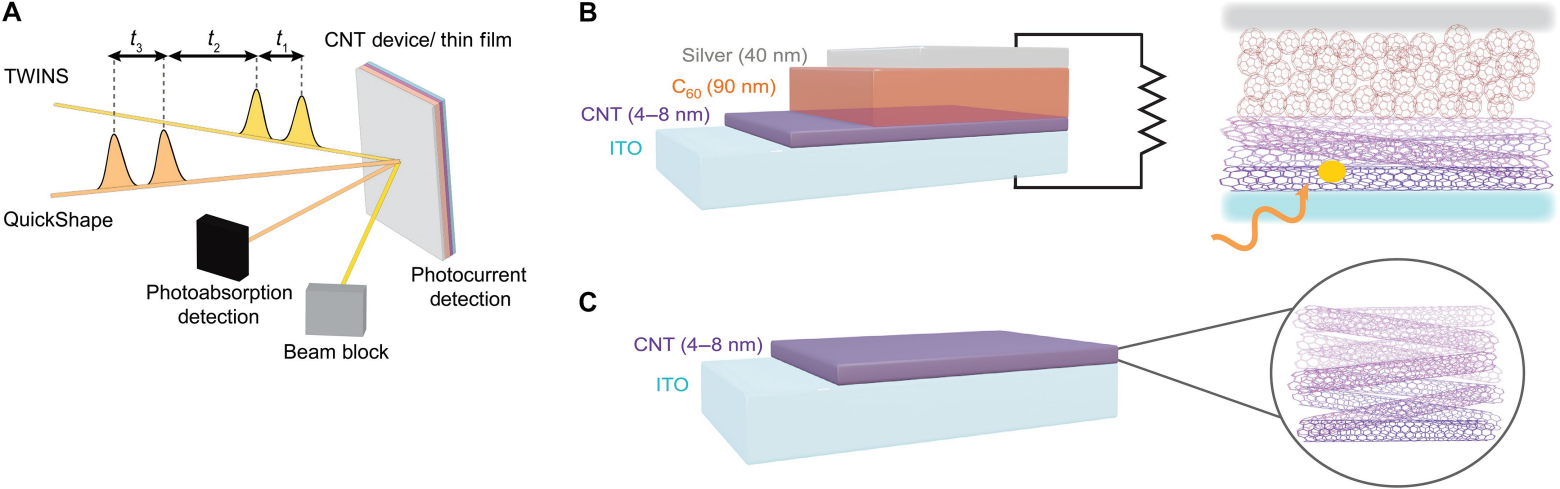
Instrument and sample schematics. (**A**) Schematic of instrument geometry used to measure simultaneous 2D photocurrent and 2D absorption spectra of CNT photovoltaic devices. The device can be replaced with a thin film, in which case only 2D absorption is measured. (**B**) Simplified diagram of a (6,5) CNT photovoltaic device. A detailed description of the device is found in Methods. (**C**) Diagram of the (6,5) thin film used in 2D absorption-only measurements.

In this study, we measure three devices and one thin film. The devices use a planar heterojunction architecture with layers of CNTs and C_60_ between an indium tin oxide (ITO) anode and a silver (Ag) cathode (Fig. 1 and Methods) (*29*) The three devices differ in their composition of CNTs, being either solely (6,5), solely (7,5), or equal portions of (6,5) and (7,5) CNTs that are mixed before deposition. The film is solely (6,5) CNTs deposited on ITO.

Photons are absorbed by the CNTs to create excitons (Fig. 1B, yellow dot). These excitons diffuse along the lengths of CNTs and hop between CNTs (*30*–*32*). Recombination occurs when the exciton reaches the end of a tube or a trap state, which exists about every 140 nm (*33*). If the exciton encounters C_60_, it can dissociate into an electron and hole. The holes travel back through the CNTs to the anode, while electrons travel through C_60_ to the cathode, resulting in current. Understanding these processes is key to using CNTs in photovoltaics.

These experiments are made possible by a new ultrafast spectrometer that can simultaneously measure photoabsorption- and photocurrent-detected spectra free from incoherent background (Fig. 1 and Methods) (*23*). As explained in Analysis and Discussion, all excitons and holes are observed with absorption detection (with holes causing half the typical bleach signal; see the Supplementary Materials), whereas only excitons and holes that ultimately produce current are measured in photocurrent detection. As we show, a comparison of absorption and photocurrent detection provides insight into exciton processes important for photovoltaic microstructure design.

### Comparison of thin-film and device dynamics

**Fig. 2.**
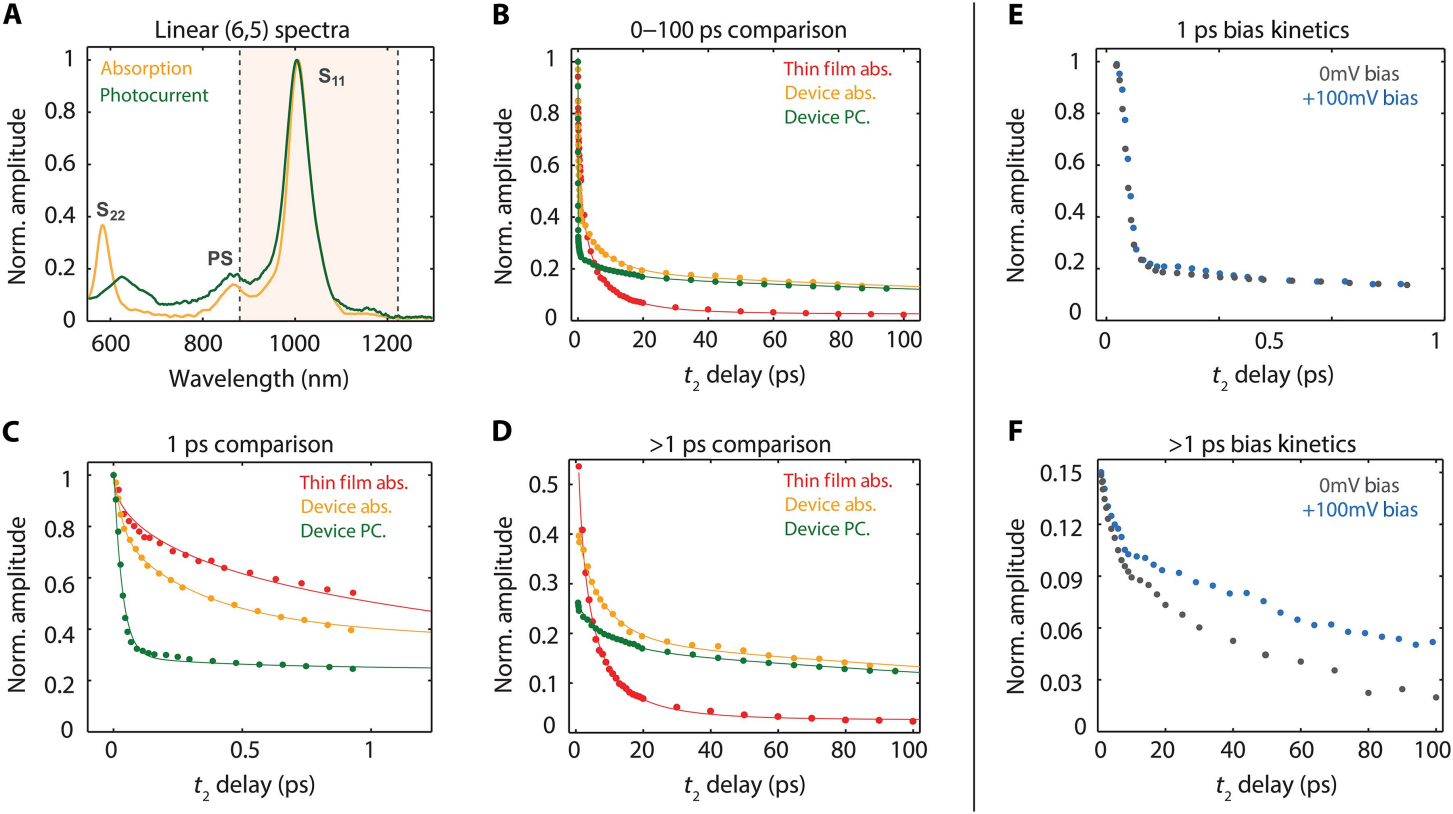
Comparison of kinetics for a (6,5) film and device measured with pump-probe photoabsorption and photocurrent detection. (**A**) Overlaid linear absorption spectrum (yellow) for a (6,5) thin film and linear photocurrent spectrum (green) for a (6,5) photovoltaic device. Three transitions are labeled: the S_11_ at 1000 nm, the phonon sideband (PS) at 860 nm, and the S_22_ at 580 nm. Because of optical interference effects within the device, only the S_11_ and PS appear strongly in the photocurrent (*55*). The shaded pink region represents the spectral width of the laser pulse. (**B** to **D**) Normalized transient absorption and photocurrent kinetics taken at 1000 nm (S_11_) for a thin film (red), an absorption-detected device (yellow), and a photocurrent-detected device (green). The solid lines in each plot are the fits described in Analysis and Discussion below. Panel (B) plots the full kinetics out to 100 ps, panel (C) plots the first picosecond of the kinetics data, and panel (D) plots *t*_2_ delays >1 ps. (**E** and **F**) Kinetics traces of unbiased (blue) and biased (gray) transient photocurrent data. Panel (E) plots the first picosecond of data, and panel (F) plots the *t*_2_ delays >1 ps. Two different photovoltaic devices were used to collect the data in (B) to (D) versus (E) and (F), which is why the relative amplitudes of their fast and slow components are slightly different.

We start by presenting data collected using films and devices made from (6,5) CNTs. Shown in Fig. 2A are the linear absorption and photocurrent spectra of a (6,5) device. The internal quantum efficiency of our devices is ~50%, meaning that half of the excitons do not generate current. The (6,5) CNTs primarily absorb at 1000 nm with phonon sideband absorption at 860 nm. These are linear spectra that do not report on exciton dynamics, so one cannot use them to determine whether excitons dissociate into charges immediately or first transfer between nanotubes to reach the C_60_ heterojunction.

Shown in Fig. 2 (B to D) are transient absorption kinetics for a (6,5) CNT film as well as kinetics for a (6,5) CNT device measured with both transient absorption and photocurrent at 1000 nm. It is apparent that each measurement exhibits different kinetics on multiple timescales. On the subpicosecond timescale (Fig. 2C), the slowest kinetics are observed in the thin film, and the fastest kinetics are observed in the photocurrent-detected device. Between 1 and 100 ps (Fig. 2D), the thin-film kinetics decay to the baseline, while the device kinetics still have ~10% of their initial intensity. Measurements for a device made from (7,5) CNTs are nearly identical to the (6,5) device (see the Supplementary Materials), which is expected because the excitons of both films will dissociate upon contact with C_60_ (*34*). No detailed analysis is needed to conclude that the kinetics in these three measurements are so different either that the photophysics of the films are altered in a device or that photoabsorption and photocurrent detection reports on different photophysics.

When excitons dissociate, they generate charges, so device measurements should contain spectroscopic signatures of both excitons and holes (*16*, *35*). To distinguish between the two, we repeated the measurements with an applied bias, given that only charges are influenced by electric fields. Shown in Fig. 2 (E and F) are kinetics measured with and without a +100-mV forward bias. The kinetic traces are normalized at *t*_2_ = 0 fs to account for the decrease in charge collection efficiency under forward bias. Before 1 ps, no notable difference in kinetics is observed with the bias. In contrast, for times after ~5 ps, the signal decays more slowly upon application of the +100-mV bias. Slower kinetics are consistent with a positive bias, which should hinder charge transport to the electrodes. Thus, the subpicosecond timescales are likely dominated by exciton dynamics, while longer timescales report on hole dynamics.

### Exciton and hole transfer between CNTs

**Fig. 3.**
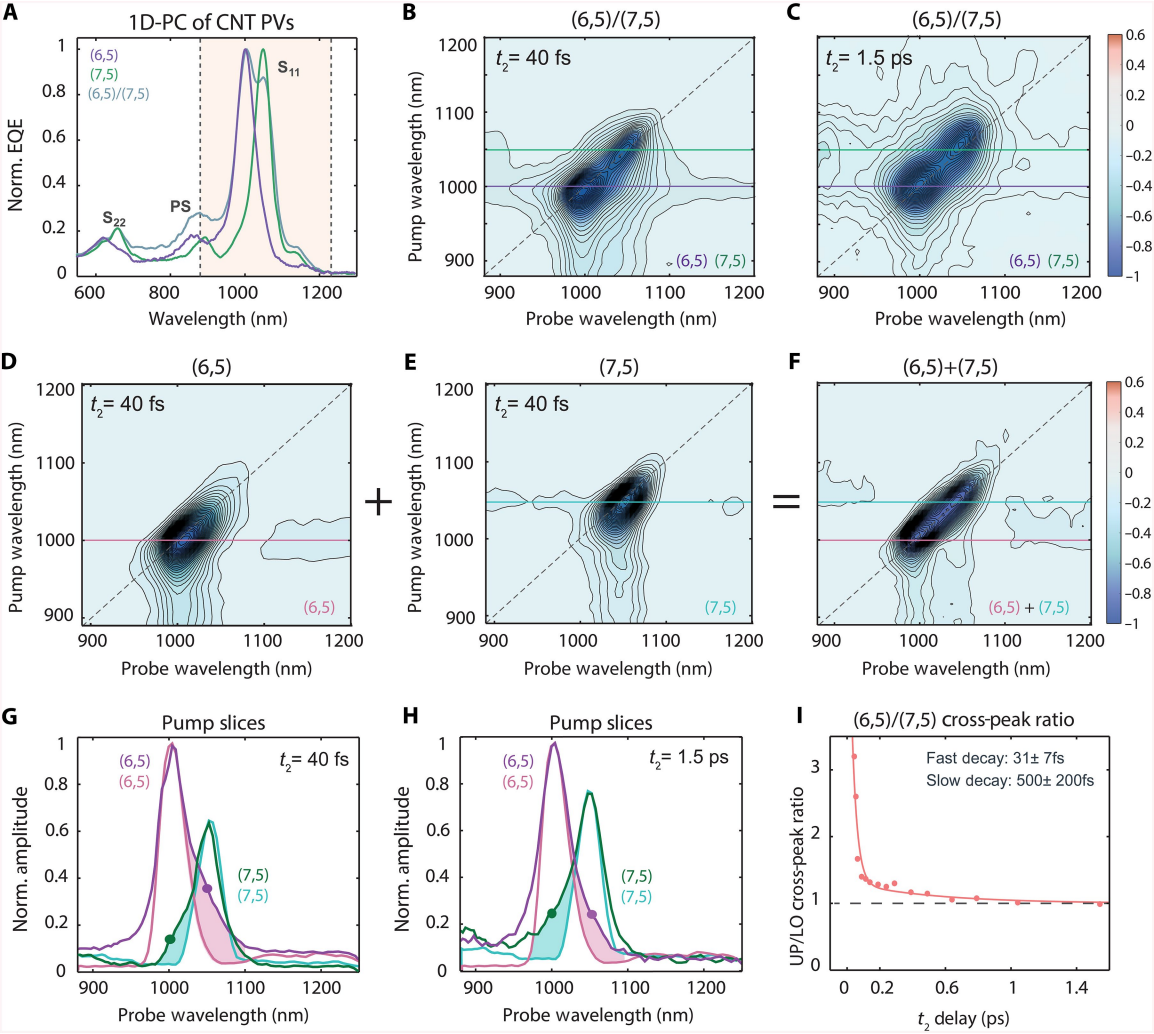
1D and 2D photocurrent-detected spectra for devices of (6,5), (7,5), and mixed (6,5)/(7,5) chiralities. (**A**) Overlapped and normalized linear photocurrent spectra of (6,5) (purple), (7,5) (green), and (6,5)/(7,5) (blue). EQE is external quantum efficiency. (**B** and **C**) Normalized 2D photocurrent spectra from a mixed (6,5)/(7,5) photovoltaic device at 40 and 1500 fs, respectively. (**D** to **F**) Process of mathematically adding single chirality spectra: Spectra of (D) (6,5) and (E) (7,5) single chirality photovoltaic devices at 40 fs are mathematically added to create (F) a (6,5) + (7,5) 2D photocurrent spectrum. (**G** and **H**) Pump slices at 1000 nm (purple and pink) and at 1050 nm (green and teal) from both the (6,5)/(7,5) mixed device and the mathematically added (6,5) + (7,5) device. Both plots are marked with purple and green dots, which represent the intensity of the upper and lower cross-peaks, respectively. Also, in both plots, the region between the mixed (6,5)/(7,5) slices and the mathematically added (6,5) + (7,5) pump slices is shaded. (**I**) Ratio of the lower cross-peak to the upper cross-peak as a function of *t*_2_ delay calculated using the intensity at the frequency of the colored points in (G) and (H). The data are fit to a biexponential decay.

The above experiments on single-chirality CNTs establish the fundamental timescales for the photophysics in these systems, but they do not resolve exciton or charge transfer between CNTs. To observe these events, we used 2D spectroscopy and devices with a photoabsorbing layer made from a mixture of (6,5) and (7,5) CNTs. Shown in Fig. 3A are linear photocurrent spectra of devices made from solely (6,5) or (7,5) nanotubes, overlaid on the photocurrent spectrum for the mixed (6,5)/(7,5) device. (7,5) nanotubes absorb at 1050 nm and are spectroscopically resolvable from (6,5) nanotubes. Cross-peaks in the 2D spectra will be created by either exciton or hole transfer, enabling experiments that monitor intertube transfer processes.

Shown in Fig. 3 (B and C) are the 2D photocurrent spectra of the mixed (6,5)/(7,5) device at *t*_2_ = 40 and 1500 fs, respectively. Two diagonal peaks are observed, corresponding to the bandgaps of the (6,5) and (7,5) nanotubes. There also exist bulges in the off-diagonal portions of the spectra where cross-peaks are expected. To assess these potential cross-peaks, we present 2D photocurrent spectra in Fig. 3 (D and E) for devices made solely of (6,5) and (7,5) CNTs, respectively. These spectra contain one predominant peak corresponding to the bandgap of either the (6,5) or (7,5) CNTs. A fictional 2D spectrum is shown in Fig. 3F, created by mathematically summing the (6,5) and (7,5) 2D spectra (Fig. 3, D and E). The summed spectrum cannot include energy or charge transfer, so it lacks cross-peaks and confirms that the features in Fig. 3 (B and C) are created by either exciton transfer, charge transfer, or both.

We also find that the cross-peak intensities are not equal on the two halves of the spectrum at subpicosecond delays. Shown in Fig. 3 (G and H) are horizontal cuts at 1000 and 1050 nm through the 2D photocurrent spectra collected at *t*_2_ = 40 and 1500 fs, respectively. The cuts are superimposed on the corresponding cuts through the mathematically summed (6,5) + (7,5) spectrum. Notice that the cross-peaks, which appear as shoulders on the sides of the larger diagonal peaks (shaded teal or pink), have unequal amplitude at *t*_2_ = 40 fs. The cross-peak below the diagonal, corresponding to downhill energy transfer, is more intense initially, but by *t*_2_ ≈ 1000 fs, the asymmetry is gone (Fig. 3, H and I). As explained in Analysis and Discussion below, both exciton and hole transfer contribute to the cross-peaks.

## ANALYSIS AND DISCUSSION

The experiments presented above include absorption detection of a film, absorption detection of devices, and photocurrent detection of devices. Upon inspection, the kinetics look very different (Fig. 2), suggesting unrelated photophysical processes. However, as we show below, the data can be fit by a unified kinetic model that includes exciton kinetics, exciton dissociation, and hole kinetics. We find that all three measurements are described by the same timescales but with each photophysical process having a different amplitude depending on the sample and type of detection.

### Exciton versus hole timescales

**Fig. 4.**
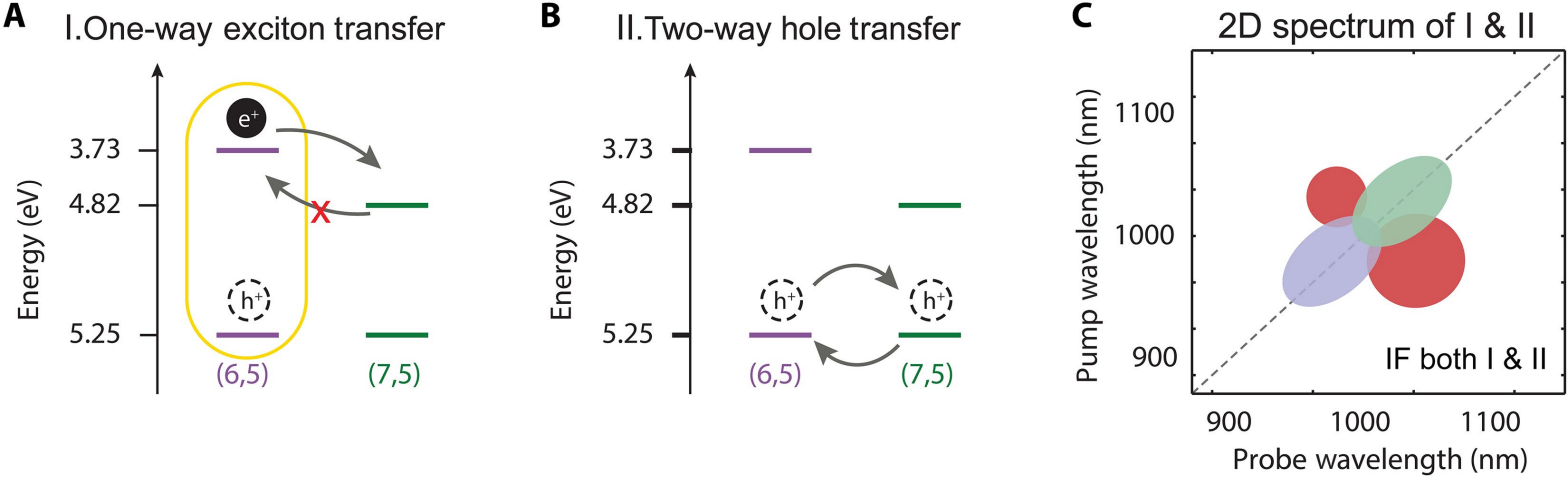
Diagram depicting the origin of asymmetric cross-peaks in 2D photocurrent spectra. (**A**) Energy level diagram for (6,5) and (7,5) CNTs with valence band maximum and conduction band minimum energy levels depicting the downhill energy transfer of excitons. (**B**) Energy level diagram depicting two-way hole transfer between (6,5) and (7,5) CNTs. (**C**) Resulting 2D photocurrent spectrum with asymmetric cross-peaks if both processes depicted in (A) and (B) are present in the system. Hole transfer can create symmetric cross-peaks, while exciton transfer only appears as a lower cross-peak.

**Fig. 5.**
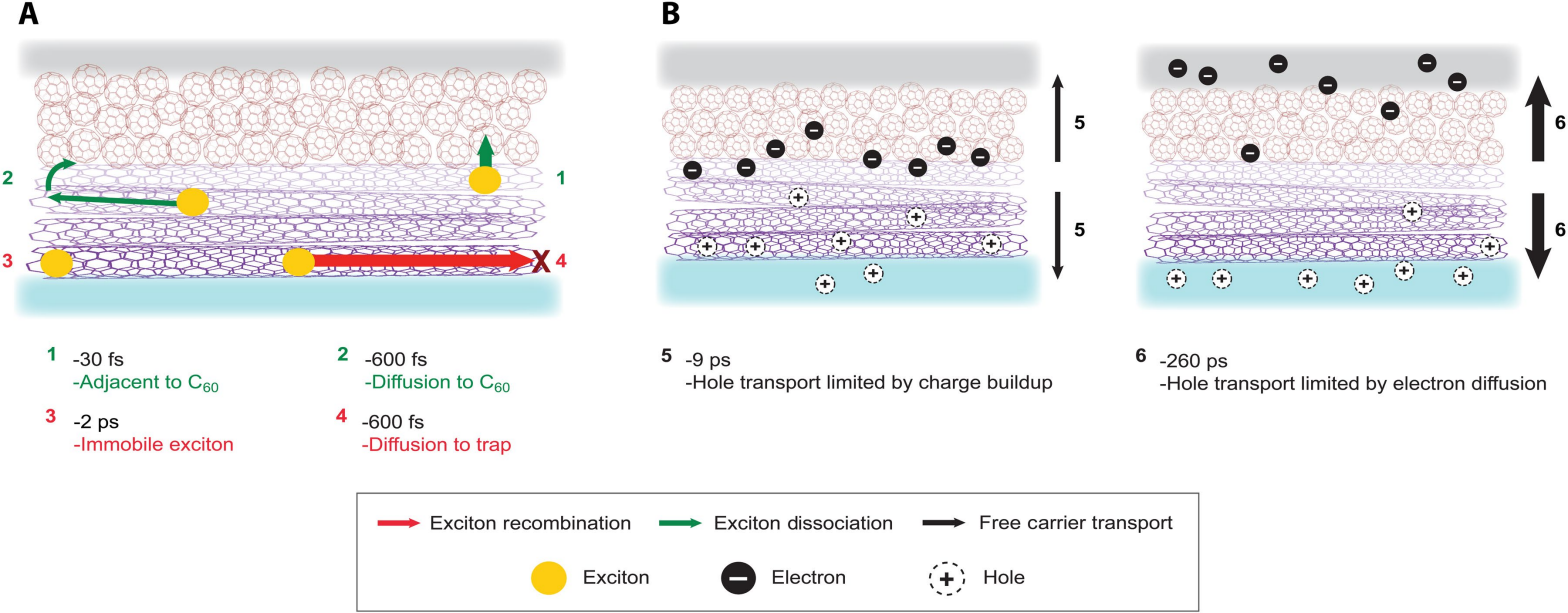
Diagrams of physical processes. These processes are listed in Table 1. (**A**) Exciton-related processes. (**B**) Carrier-related processes. The color of the arrows corresponds to exciton recombination (red), exciton dissociation (green), and carrier transport (black). The size of the arrows is proportional to the relative amplitude of the depicted process.

A key aspect of interpreting these transient photocurrent and absorption kinetics is the assignment of timescales associated with excitons versus holes. To do so, we start by interpreting the 2D photocurrent data in Fig. 3. Shown in Fig. 4 (A and B) are the energy levels of the valence and conduction bands at *k* = 0 momentum for (6,5) and (7,5) CNTs. The difference in bandgap between the (6,5) and (7,5) CNTs is much larger than *k*_b_*T* (where *k*_b_ is the Boltzmann constant, and *T* is the temperature), so exciton transfer can only occur downhill in energy from (6,5) to (7,5) CNTs (Fig. 4A), creating a cross-peak on the lower half of the spectrum. If the cross-peaks were solely due to exciton transfer, no cross-peak would be observed on the upper half of the 2D spectrum. The bandgaps of (6,5) and (7,5) CNTs are different because of the energies of their conduction bands, but their valence bands are nearly degenerate (*34*). As a result, hole transfer can occur from (6,5) to (7,5) tubes and vice versa, creating cross-peaks on both sides of the diagonal with equal magnitude. Thus, upper and lower cross-peaks with a more intense lower cross-peak is a clear signature that exciton and hole transfer occurs (Fig. 5C). With this assignment, we fit the ratio of the cross-peak amplitudes (Fig. 3I) to a biexponential decay, giving time constants of 31 ± 7 and 500 ± 200 fs for exciton transfer from (6,5) to (7,5) CNTs. At *t*_2_ ≈ 1 ps, the cross-peaks have equal intensities (Fig. 3, C and H, until ~30 ps; see the Supplementary Materials). Thus, after 1 ps, excitons that have transferred between tubes no longer contribute to the photocurrent, and only hole transfer occurs between CNTs.

### Unified model for a global fit of kinetics

To fit the transient photocurrent and absorption kinetics of the (6,5) single chirality device in Fig. 2, we present the following model. The recombination rate of excitons is modeled by a stretched exponential (Eq. 1), following prior work linking the exciton lifetime to defect densities (*28*, *33*). For all other photophysical processes, such as exciton dissociation and hole transfer, we assume first-order rate equations (Eqs. 2 and 3)

$$\frac{dP_{\text{recom}}\left(t\right)}{\mathrm{dt}}=-\frac{1}{2a}*{\left(\frac{t}{a}\right)}^{-1/2}*P_{\text{recom}}\left(t\right)$$ (1)

$$\frac{dP_{\text{dis}}\left(t\right)}{\mathrm{dt}}=-b*P_{\text{dis}}\left(t\right)-c*P_{\text{dis}}\left(t\right)$$ (2)

$$\frac{\mathrm{dH}\left(t\right)}{\mathrm{dt}}=b*P_{\text{dis}}\left(t\right)+c*P_{\text{dis}}\left(t\right)-d*H\left(t\right)-e*H\left(t\right)$$ (3)

In the above equations, *P*_recom_, *P*_dis_, and *H* represent the populations of excitons that recombine, excitons that dissociate, and holes, respectively. *a* to *e* are the time constants for each process. To calculate the population of excitons as a function of time, we integrate the rate equations (see the Supplementary Materials). Note that because our fits use integrated rate equations, the relative amplitude of each process, not the integrated area, is proportional to the photocurrent it will generate (see the Supplementary Materials). We find that a minimum of four first-order rate equations is necessary to fit the data (two for Fig. 2C and two for Fig. 2D). We also note that according to the voltage-dependent kinetics (Fig. 2, E and F) and the asymmetric cross-peaks (Fig. 3I), only holes contribute to the photocurrent after ~1 ps. Thus, two of the rate equations are assigned to excitons and two to holes. Given that the dissociation of an exciton is required to create a hole, these two sets of equations are coupled. The resulting amplitudes and time constants are used to simulate the kinetics (see the Supplementary Materials), noting that not all photophysical processes contribute to each measurement. The dominant photophysical process in the films is the exciton lifetime (Eq. 1), given that only a very small percentage of excitons dissociate in films (see the Supplementary Materials) (*36*). In contrast, the exciton lifetime does not contribute to the photocurrent data because photocurrent is not generated from excitons that recombine. All five physical processes are included in the device transient absorption experiments because absorption is sensitive to both excitons and holes regardless of whether they contribute to the photocurrent.

Using this model, we globally fit the kinetic data in Fig. 2 from 0 to 100 ps by constraining the time constants to be the same across measurements while allowing relative amplitudes to vary. The fits are excellent, and the resulting amplitudes and time constants are given in Table 1. We find that each photophysical process contributes a different amount to each of the three measurements. For example, 30-fs exciton dynamics dominate the photocurrent kinetics (89%), contribute moderately to the photoabsorption kinetics of the devices (33%), and do not contribute to film kinetics. As a result, the kinetic traces appear to be disparate, but the transient absorption and transient photocurrent data actually contain the same photophysics. The same global fit procedure is applied to a (7,5) single chirality device in the Supplemental Materials, and we obtain similar results. We emphasize that the ultrafast dynamics reported here are not indicative of photocurrent being generated on a 30- or 600-fs timescale; rather, the timescales report on processes that eventually result in current generation.

**Table 1.** Time constants and relative amplitude of the photophysical processes observed in the kinetics data in Fig. 2. Not all physical processes are present for each type of sample or mode of detection. The amplitudes for the populations of the excitons and holes are normalized separately. The reported errors in time constants and amplitudes are the standard errors calculated on the basis of the sum of square residuals and the degrees of freedom in the fit. See the Supplementary Materials for additional details. The rows labeled Productive abs. and Unproductive abs. are subsets of the optically detected fit calculated on the basis of the relative intensities of the fast and slow exciton processes in the photocurrent-detected fit. Productive abs. represents the portion of the optically detected excitons that generate current, and Unproductive abs. represents the portion of the optically detected excitons that do not generate current. Because of these devices’ high charge collection efficiency, we assume that all charges are productive.

Time constants	2 ± 0.1 ps	30 ± 3 fs	600 ± 100 fs	9 ± 1 ps	260 ± 20 ps
Film abs.	1.0	0	0	0	0
Device current	0	0.89 ± 0.02	0.11 ± 0.01	0.27 ± 0.03	0.73 ± 0.03
Device abs.	0.21 ± 0.02	0.33 ± 0.03	0.46 ± 0.03	0.21 ± 0.03	0.79 ± 0.04
Productive abs.	0	0.33	0.04	0.21	0.79
Unproductive abs.	0.21	0	0.42	0	0
Assignment	Recombination	Adjacent to C_60_	Diffusion	Fast hole collection	Slow hole collection

### Physical interpretation of the photophysics and deductions for device design

The fits give an exciton lifetime of 2 ps, exciton dynamics of 30 and 600 fs, and hole dynamics of 9 and 260 ps (Table 1). There exists a large body of work on CNT exciton dynamics that we draw upon to provide a physical interpretation of these timescales (graphically illustrated in Fig. 5) (*10*, *28*, *30*–*32*, *37*–*39*). First, the 2-ps exciton lifetime is typical for films made from CNTs of similar lengths and defect density (*28*, *32*). While diffusing along CNTs (at ~750 nm^2^/ps), excitons encounter defects in the tube walls and ends, causing recombination (*28*, *31*). 750 nm^2^/ps is so fast that the exciton lifetime is mostly set by the distance between defects. Second, we assign the 30- and 600-fs timescales to dissociation of excitons to charges by C_60_. These timescales are not observed in the (6,5) film and so must be caused by exciton dissociation. On the basis of the in-plane (lateral) diffusion constant mentioned above, excitons can travel at most ~4 nm in 30 fs, so these excitons are formed adjacent to or nearby C_60_. Third, we assign the 600-fs timescale to excitons that must appreciably diffuse or first transfer to another CNT. Regarding the 600-fs timescale, we know from prior work that energy transfer between CNTs occurs at intersections of crossing tubes (*10*, *32*). At the densities of these films, it takes an exciton an average of 500 fs to diffuse to a suitable intersection (*32*, *38*). Moreover, we know for certain that exciton transfer before dissociation contributes to the photocurrent because the cross-peaks in Fig. 3B exhibited a similar 500-fs timescale. Therefore, we assign the 600-fs timescale to excitons that must appreciably diffuse or first transfer to another CNT before dissociating. We note that the (6,5)/(7,5) cross-peak data also had a 30-fs timescale, meaning that the photocurrent is generated both by excitons immediately transferring between parallel tubes and by excitons first diffusing to crossing points (*37*).

As established by our experiments, the 9- and 260-ps timescales are associated with holes. Less is known about hole transfer in our CNT devices. We tentatively assign the two timescales to fast charge buildup at the anode and slower transport through the CNT layer (Fig. 5). The relative amplitude and timescales of these kinetics will also be influenced by the rate of electron collection, given that the device must maintain neutrality. Hole dynamics do not affect efficiency in these devices because charge transfer excitons do not form between CNTs and C_60_, and all charges will inevitably be collected (*40*). Therefore, we leave the precise interpretation of these timescales for a future study.

We can also estimate the internal quantum efficiency of the device at this spot from our fits by comparing the relative amounts of exciton dissociation versus recombination. The exciton timescales are 30 fs, 600 fs, and 2 ps (the others are holes). The 2-ps timescale is solely recombination, given that it does not appear in the photocurrent. The ratio of exciton dissociation in the 30- and 600-fs timescales must be the same in photocurrent and optical detection, given that they are on the same device at the same location. If the 30-fs time constant is solely due to dissociation, then only ~10% of the 600-fs process is productive, and the internal quantum efficiency of the measured spot is 37%. Table 1 summarizes the calculation. The remainder of the 600-fs optically detected signal (42%) must be from unproductive processes that occur on a similar timescale to exciton diffusion to C_60_.

### Summary and implications for device design

The photocurrent experiments presented here determine that 89% of the current derives from excitons that are harvested within 30 fs of their generation (Table 1, row 3). These excitons are generated adjacent to or nearby C_60_. Only 11% of the photocurrent is created by excitons that find the C_60_ interface within 600 fs via diffusion and hopping. After about 1 ps, excitons continue to transfer between CNTs but no longer generate current. The exciton lifetime of 2 ps is much longer than these timescales. Thus, even though excitons continue to exist in the films for extended periods of time, most do not contribute to the current. These conclusions are further supported by the fact that internal quantum efficiency decreases with increasing CNT thickness (*41*). As the CNT layer becomes thicker, fewer CNTs will be in close contact with C_60_, making the 30-fs dissociation process responsible for the majority of current generation less likely.

This previously unknown information markedly affects our thinking for how best to design CNT photovoltaics. On the basis of transient absorption spectroscopy of films, our efforts have been focused on improving exciton transfer. Toward that end, film morphologies were tested that changed the distance between tube intersections, causing a corresponding change in exciton transfer (*10*, *38*). Exciton transfer between CNTs was improved 10-fold by removing residual polymer wrapping, enabling more direct CNT contact (*37*). Defect densities were modified to extend exciton lifetimes (*28*). These experiments led to a better understanding of excitons in these films but did not significantly improve device efficiency. These results now make sense knowing that the vast majority of current is generated from excitons that dissociate within 30 fs. An alternative direction would be leveraging the 30-fs process such as by intercalating the C_60_ into the CNT layer. CNT/C_60_ contact might also be improved by better disaggregating the CNTs, which bundle into fibrils of ~7 to 19 tubes (*38*).

The default approach for assessing exciton dynamics is transient absorption of thin films. That approach relies on the assumption that the exciton dynamics deduced in films are reflected in the current-generating pathways of the corresponding device. As we show here, that assumption is a poor approximation for CNT photovoltaics. It will be interesting to apply this new spectrometer design to other photovoltaic materials to assess whether the conclusions drawn from transient absorption hold true in those working devices.

## METHODS

### Spectrometer

The pump-probe and 2D spectrometer (Fig. 1 and the Supplementary Materials) that can simultaneously measure photoabsorption and photocurrent with no incoherent background has been described in detail elsewhere (*23*). It consists of an ytterbium laser operating at a 100-kHz repetition rate that pumps an optical parametric amplifier to make 800-nm pulses, followed by white light (850 to 1300 nm) generation using yttrium-aluminum-garnet supercontinuum generation (*42*). A pair of pump pulses separated by a delay *t*_1_ is created using a TWINS interferometer configured to create orthogonally polarized pulses oriented at 0° and 90° in the lab frame (*43*–*45*). A pair of probe pulses separated by a delay *t*_3_ is created using a transverse, acousto-optic modulator–based pulse shaper (Phasetech Quickshape), allowing shot-to-shot modulation of time delays and phases at the 100-kHz repetition rate (*46*–*50*). A waveplate and a polarizer are placed after the pulse shaper to ensure that the probe pulses are oriented at 45°. A motorized delay stage sets the relative delay between the two sets of pulse pairs, *t*_2_. With this configuration, four pulses with variable delays (*t*_1_, *t*_2_, and *t*_3_) are created with pulse polarizations of <0°,90°,45°,45°> that eliminates the incoherent mixing background in these samples. Both the pump and probe pulses have low pulse energies (∼200 pJ) with a spot size of ~150 μm at the sample, which is estimated on the basis of the diameter of the beam entering our focusing optic (1.5-mm beam entering a 200-mm-focal-length off-axis parabolic). These measurements correspond to a pump incident fluence of 1.1 × 10^12^ photons/cm^2^. The absorbed pump fluence is ~4.0 × 10^11^ photons/cm^2^, which corresponds to an initial exciton density of ~1.25 excitons/CNT (see the Supplementary Materials for calculations). Note that the absorbed fluence is an approximation based on typical losses from device layers other than the active layer, which is ~30%. The initial bleach signal scales linearly with respect to pulse power, and the kinetics do not change with increasing or decreasing fluence (see the Supplementary Materials).

When acquiring 2D spectra, the signal intensity is measured as a function of both *t*_1_ and *t*_3_ delays, and a 2D Fourier transform is calculated to obtain the pump and probe frequency axes, respectively. In each experiment, we implement a four-frame phase cycle using the pulse shaper. For pump-probe experiments, we set *t*_1_ = 0 so that there is effectively a single pump pulse and collect the signal as a function of the *t*_3_ delay, thereby resolving the probe frequencies with a Fourier transform. For pump-probe measurements, we also implement an additional pseudophase cycle by modulating the pump pulses’ relative time delay by half of an optical cycle. To measure population dynamics, spectra are collected as a function of *t*_2_ delay. By measuring the probe light reflected off the sample and onto a single pixel detector, we simultaneously acquire nonlinear photoabsorption and photocurrent signals at the same sample position. The thin-film measurements are also performed in reflection.

Because of the low white-light fluence, the time resolution of the spectrometer is obtained using polarization-gated frequency-resolved optical gating (PG_FROG) with a separate 800-nm pulse (130 ± 2 fs). The pump and probe pulses are 17 ± 4 and 29 ± 5 fs in duration, respectively, assuming Gaussian time profiles, giving an instrument response time of 33 ± 6 fs.

### Samples

The three devices use a stacked architecture consisting of 40-nm poly(3,4-ethylenedioxythiophene) polystyrene sulfonate on an ITO substrate (anode) as an electron-blocking material, a photoactive CNT layer deposited via drop casting, 90-nm C_60_, 10-nm hole-blocking material bathocuproine, and 120-nm Ag for the cathode. See the Supplementary Materials for more details. Each one is encased using epoxy and a glass coverslip. The three devices differ in the mixture and thicknesses of the CNT layer: a 4-nm-thick layer composed solely of (6,5) CNTs, a 4-nm-thick layer composed solely of (7,5) CNTs, and an 8-nm-thick layer containing equal portions of (6,5) and (7,5) CNTs that are mixed before depositing. We also performed measurements on a 4-nm-thick film of (6,5) CNTs deposited on ITO via drop casting in an identical manner as the devices (Fig. 1C). The (6,5) and (7,5) CNTs used in these samples were purified using shear force mixing and aromatic polymers, respectively (*51*, *52*).

We avoid coffee rings during our drop-casting process by drying our CNT-containing *ortho*-dichlorobenzene solution slowly over 48 hours for a 1.5 cm–by–2.0 cm substrate. A photograph of a smooth, continuous film made with this method is available in our previous work (*53*). The films have a root-mean-square surface roughness of 2 to 4 nm [see atomic force microscopy by Dhavamani *et al.* (*53*) and Blackwell *et al*. (*54*)].
