# Supplementary material for: Ultrafast photocurrent detection contradicts optical detection conclusions: Exciton diffusion contributes little to carbon nanotube device efficiency
Source: Sci Adv. 2026 Jul 1;12(27):eaec3186. doi: 10.1126/sciadv.aec3186 (PMC13322258; doi:10.1126/sciadv.aec3186)
Supplement: Supplementary file 1 — Supplementary Text Figs. S1 to S8 Table S1 [file sciadv.aec3186_sm.pdf]

Science Advances
AAAS

Supplementary Materials for

Ultrafast photocurrent detection contradicts optical detection conclusions: Exciton diffusion contributes little to carbon nanotube device efficiency

Zachary M. Faitz *et al.*

Corresponding author: Zanni@chem.wisc.edu

Sci. Adv. **12**, eaec3186 (2026)
DOI: 10.1126/sciadv.aec3186

This PDF file includes:

Supplementary Text
Figs. S1 to S8
Table S1

Supplementary Figures

Supplementary Figure 1: Signal Intensity vs. Pump Power

We show that the signal intensity of the S_{11} feature in a 2D photocurrent spectrum of (6,5) CNT photovoltaic device is linear with pump power. We chose a (6,5) device to test power dependence because the peak intensity of our pump spectrum is at 1000nm and (6,5) absorbs at 1000nm. All dynamics in the work were measured using a pump power of 20μW. This figure also includes the pump fluence dependence of optical- and photocurrent-detected kinetics. We observe no change in the measured kinetics across the chosen pump fluences. These fluence dependent measurements were performed with less averaging than the other measurements in this paper to make the data acquisition time faster. This allows us to avoid long term fluctuations in power and obtain accurate power measurements.

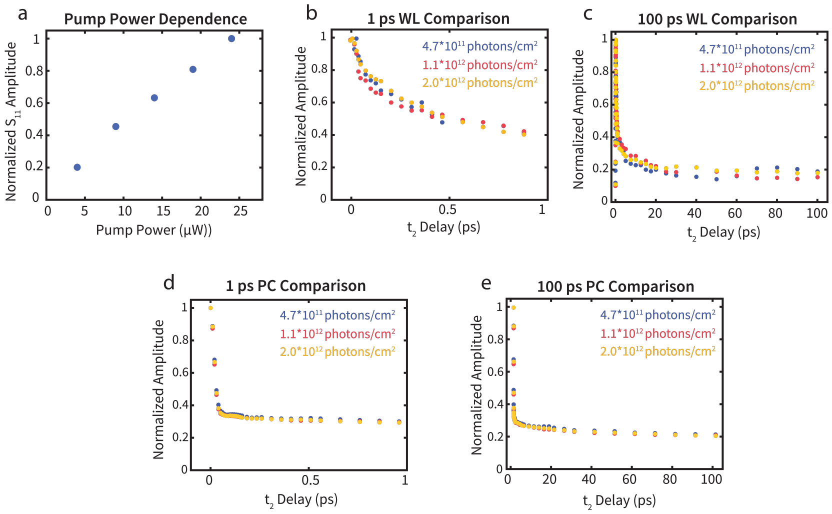

Supplementary Fig. 1: Measurements showing the CNT photovoltaic devices behave linearly under the pump power and fluence conditions used in this work. (a) A plot of the S_{11} bleach amplitude from a (6,5) CNT photovoltaic device versus pump power. The measured amplitudes are normalized to the amplitude at 25μW. The data in the main text was collected using a pump power of 20μW. (b,c) Transient absorption kinetics of a (6,5) device measured under a range of fluences. (b) shows the first ps of transient absorption kinetics and (c) shows the full 100 ps of transient absorption kinetics.(d,e) The corresponding transient photocurrent kinetics to the transient absorption measurements in (b,c). (d) shows the first ps of transient photocurrent kinetics and (e) shows the full 100ps of transient photocurrent kinetics. Note that the data in (b-e) were measured using a different (6,5) device than the data in the main text; however, the two devices were prepared on the same substrate, so the individual layers and performance are nearly identical to the device used in the main text

Supplementary Figure 2: Carriers vs. Monochromatic Power

We show that the number of carriers generated in a (6,5) CNT photovoltaic device is linear with monochromatic pulsed light power. Our monochromatic light was created by placing a 1000 ±5nm bandpass filter in the white light pump. We calculate the number of carriers generated by integrating over the signal produced on an oscilloscope. Other fabrication-focused works report that the number of carriers generated in CNT-based Devices is linear with monochromatic light power until $\sim 3\ \mu$W of 1000 ±5nm. Measurements are performed at 0.9 μW of 1000 ±5nm, which corresponds to the lowest point on the power dependent curve. This is evidence that we are studying our devices in a regime where they behave normally.

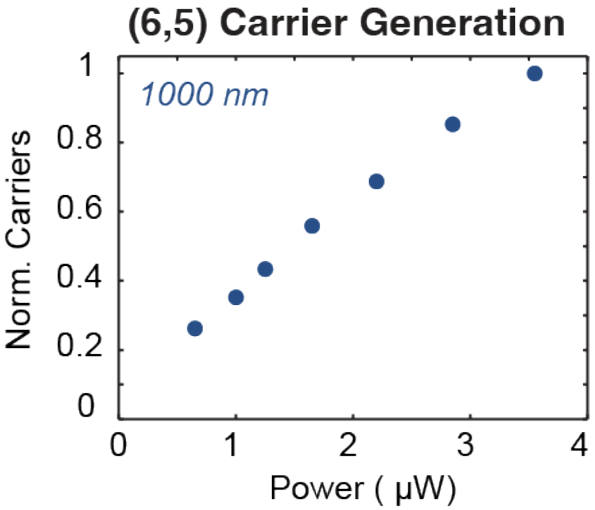

Supplementary Fig. 2: Depicts the normalized amount of carriers generated as a function of 1000nm power. The number of carriers is normalized to the carriers generated with 3.5μW of 1000nm light.

Supplementary Figure 3: Detection of Excitons and Carriers

By comparing the pump-off and pump-on diagrams in Supplementary Fig. 3 below, one can understand the bleach amplitude of the system at various t_2 delays. The amplitude of the bleach is calculated by subtracting the pump-off diagram from the pump-on diagram. From these diagrams we can see that photocurrent spectroscopy is unable to report on trap states or pathways that end in recombination because there is no change in the photocurrent when the pump is on or off. We note that the holes themselves are not optically active and so do not absorb the probe. Nonetheless, they contribute features to the spectra because the excitons that dissociated to create those holes leave a bleach. Since they create a bleach but not stimulated emission, they only contribute half the signal intensity. Holes can be monitored by probing the trion band, but that wavelength is outside of those studied here.

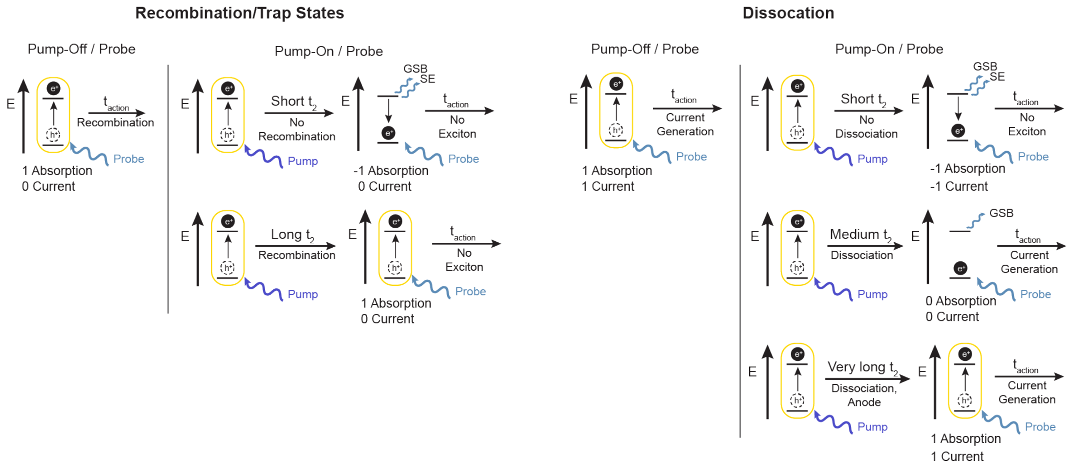

Supplementary Fig. 3: Diagrams for calculating the bleach amplitude at various t_2 delays for recombination and dissociation pathways. The listed amplitude is calculated by subtracting the absorption or photocurrent of the pump-off diagram from the absorption or photocurrent of the pump-on diagram.

Supplementary Figure 4: (7,5) Transient Photocurrent Kinetics

In Supplementary Fig. 4 we report the transient photocurrent and transient absorption kinetics of a (7,5) CNT photovoltaic device. These measurements are analogous to the (6,5) transient photocurrent kinetics reported in the main text This device was prepared using the same methods as the (6,5) device; however, its internal quantum efficiency is ∼56% rather than ∼37% based on the method of calculating the relative amounts of productive and unproductive excitons in the optically detected device measurements described in the main text. Supplementary Fig. 4a contains both the linear absorption and photocurrent of a thin film and a device. Supplementary Fig. 4b-d are the transient absorption and transient photocurrent kinetics spanning 0 to 100 ps measured at 1050nm. Similarly to the (6,5) data in the main text, the fit parameters are summarized in the table below.

Time Constants	2 ±0.1ps	34 ±3fs	500 ±100fs	7 ±1ps	260 ±20ps
Device Current	0	0.88 ±0.01	0.12 ±0.01	0.39 ±0.02	0.61 ±0.03
Device Abs.	0.1 ±0.02	0.49 ±0.03	0.41 ±0.05	0.35 ±0.03	0.65 ±0.04
Productive Abs.	0	0.49	0.07	0.35	0.65
Unproductive Abs.	0.1	0	0.34	0	0
Assignment	Recombination	Adjacent to C_{60}	Diffusion to C_{60}	Fast Hole Collection	Slow Hole Collection

Supplementary Table 1: Time constants and relative amplitudes of the photophysical processes observed in the kinetics data in Supplementary Fig. 4. Not all physical processes are present for each type of sample or mode of detection. The amplitudes for the populations of the excitons and holes are normalized separately. The reported errors in time constants and amplitudes are the standard errors calculated based on the sum of square residuals and the degrees of freedom in the fit.

We first note that the time constants measured in the (7,5) sample are the same as the (6,5) sample within error. This is expected since the binding energy of (7,5) excitons is roughly the same as (6,5) excitons (∼200meV), and the difference between the CNT conduction band and the C_{60} LUMO is much greater than 200meV. Differences arise between the (6,5) and (7,5) samples, however, when we examine the amplitudes of these time constants. For example, the exciton recombination process has less than half the amplitude in the (7,5) device when compared to the (6,5) device. Additionally, we find that the ∼30fs exciton dissociation constant increases in relative amplitude when comparing the (6,5) and (7,5) devices. These differences are a strong indication that are assignments to these time constants are correct given that the (7,5) device is ∼20% more internally efficient than the (6,5) device. As internal quantum efficiency increases, we would expect the relative amplitude of the least efficient process (recombination) to decrease while the relative amplitude of the most efficient process (exciton dissociation adjacent to C_{60}) to increase.

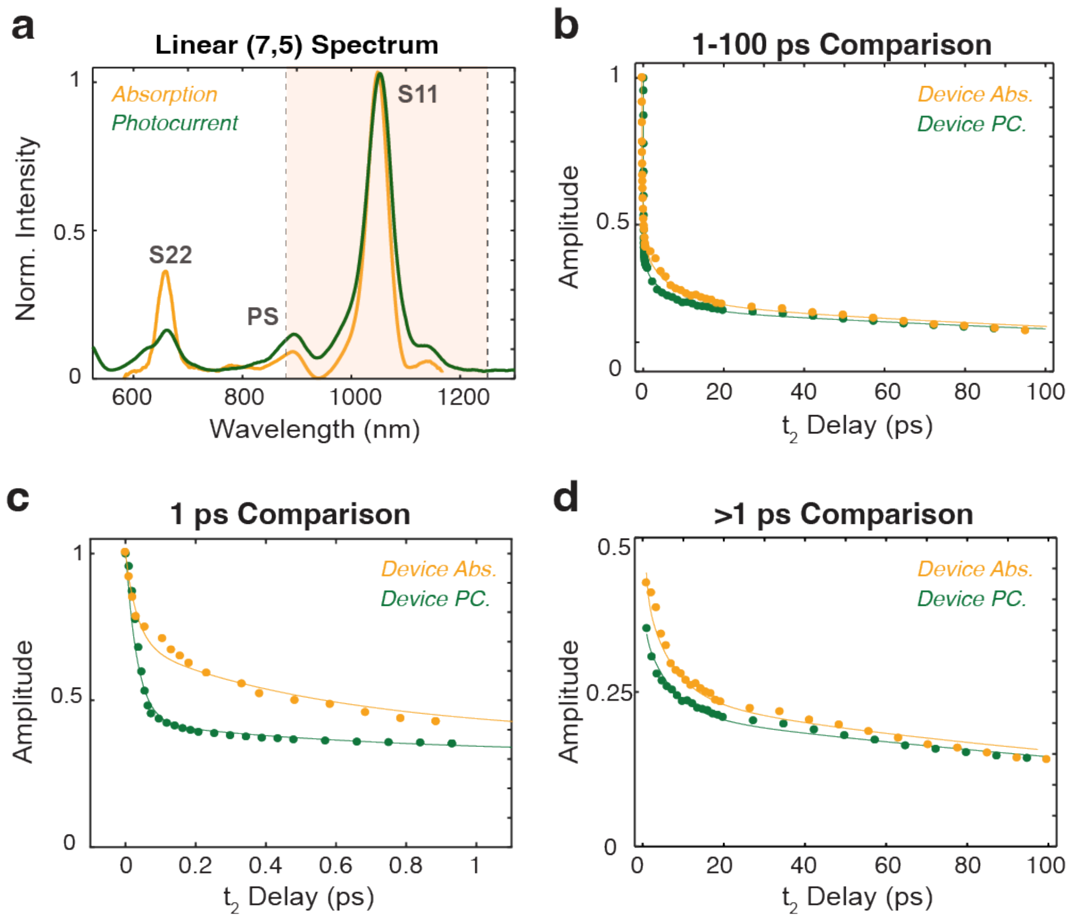

Supplementary Fig. 4: Depicts linear spectra and kinetics data for a (7,5) CNT photovoltaic analogous to Fig. 2 in the main text. (a) Overlayed linear absorption (yellow) and photocurrent (green) spectra for a (7,5) photovoltaic. Three transitions are labeled: the S_{11} at 1050nm, the phonon sideband (ps) at 900nm, and the S_{22} at 630nm. The shaded pink region represents the spectral width of the laser pulse. (b-c) Normalized transient absorption and photocurrent kinetics taken at 1050nm (S_{11}) for an absorption-detected device (yellow) and a photocurrent-detected device (green). (b) plots the full (7,5) kinetics out to 100ps, (c) plots the first 1ps of the kinetics data, and (d) plots t_2 delays greater than 100ps.

Supplementary Figure 5: Additional Examples of 2D-PC Spectra

In the figure below we report additional 2D-PC spectra of (6,5) single chirality, (7,5) single chirality, and (6,5)/(7,5) mixed chirality of CNT photovoltaic devices taken at different sample positions from the spectra in the main text. We emphasize that while the presence of the spectra features are consistent across different sample positions, the relative intensity of many features change, suggesting some level of sample inhomogeneity on the scale of out beam size (~150 microns). We highlight a few the most prominent differences. In Supplementary Fig. 5a,b, we find the lower cross peaks involving the minority chirality (9,7) (950nm) and the (7,5) phonon sideband (900nm) are more resolved. In several spectra (Supplementary Fig. 5a,d,c) the broad lower cross peak on the right side of the spectra is more intense, and, in the case of Supplementary Fig. 5a, there appear to be resolved peaks with frequencies corresponding to (11,2) (1120nm) and the (6,5) trion (1160nm). In Supplementary Fig. 5a,f, an broad upper cross peak is visible above the S_{11} transitions. This additional evidence that a trion transition is involved since it too shares a ground state with S_{11}, creating a characteristic cross peak pattern similar to the S_{11} and phonon sideband. The single chirality device diagonal peaks in Supplementary Fig. 5a,b,e appear exceptionally broad, likely because more minority chirality is present at these spots. The best example is Supplementary Fig. 5b, where the (9,7) minority chirality diagonal peak and cross peak are both visible at 950nm. The large variation in minority chirality concentration suggests than in the fabrication process minority chirality CNTs clump together. Finally, we note that in Supplementary Fig. 5c, the (7,5) S_{11} peak appears relatively weak and not well resolved from the (6,5) S_{11} despite not being a minority chirality. Furthermore, the bulge-like feature that appears at the (6,5)/(7,5) cross peak position in the Supplementary Fig. 5f and Figure 3b in the paper seems absent in Supplementary Fig. 5c, suggesting that less hole transfer and exciton transfer is taking place at this sample position.

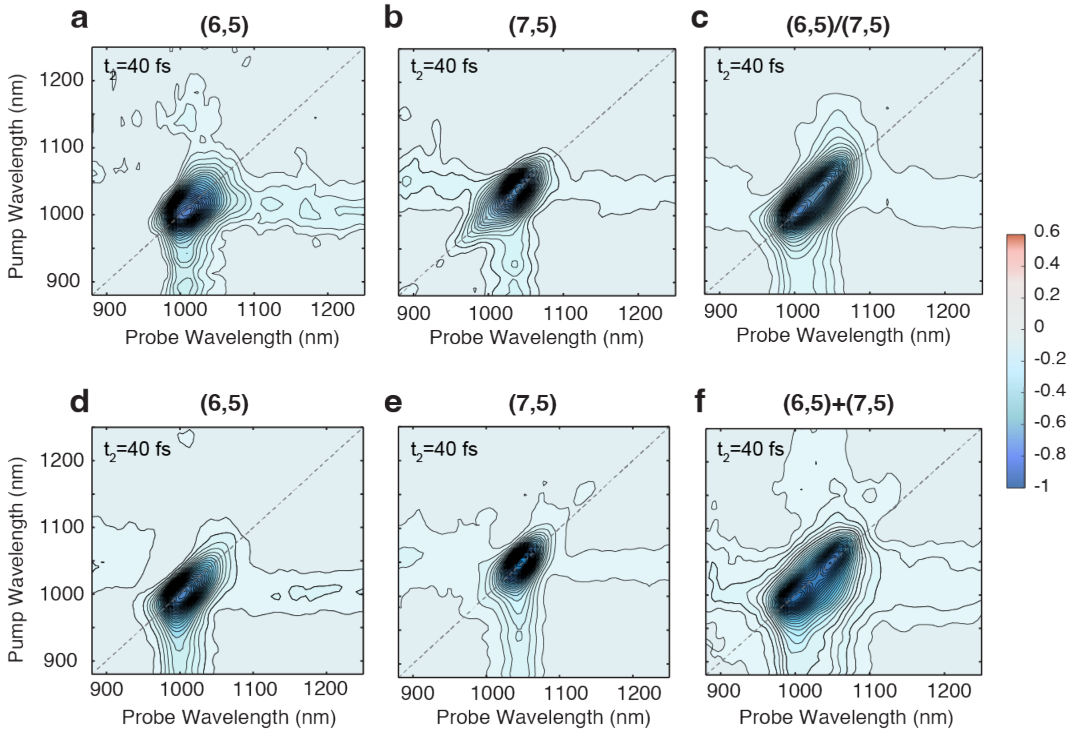

Supplementary Fig. 5: Depicts additional, normalized 2D photocurrent spectra of (a,d) (6,5) single chirality device, (b,e) (7,5) single chirality device, and (c,f) (6,5)/(7,5) mixed chirality device at different sample positions. Each Spectra is normalized to its most intense feature.

Supplementary Figure 6: Comparison of 2D-PC and 2D-WL Spectra

In this figure, we compare the 2D photocurrent spectra reported in the main text with their corresponding 2D white light spectra. Supplementary Fig. 6a,d come from the (6,5) single chirality device. Supplementary Figure 6b,e come from the (7,5) single chirality device. Supplementary Fig. 6c,f come from the (6,5)/(7,5) mixed chirality device. Each pair of spectra we collected at the same time. Most features are conserved between spectra. For example, diagonal peaks corresponding to the CNT chiralities present in the sample appear in both photocurrent and white light spectra. Moreover, features indicative of minority chiralities, such as the elongated diagonal in the (6,5) spectra and the lower cross peaks are also present in the white light spectra; although, they have lower relative intensities compared to their photocurrent counterparts. The biggest difference within the pair of spectra is the presence of an excited state absorption feature in the 2D white light spectra. This feature is not present in the 2D photocurrent spectra (and 2D action spectra in general) due to the cancellation of Feynman pathways. The lack of an excited state absorption feature in 2D action spectra allows for more accurate determination of lineshapes as well as the identification of upper cross peaks like in this paper.

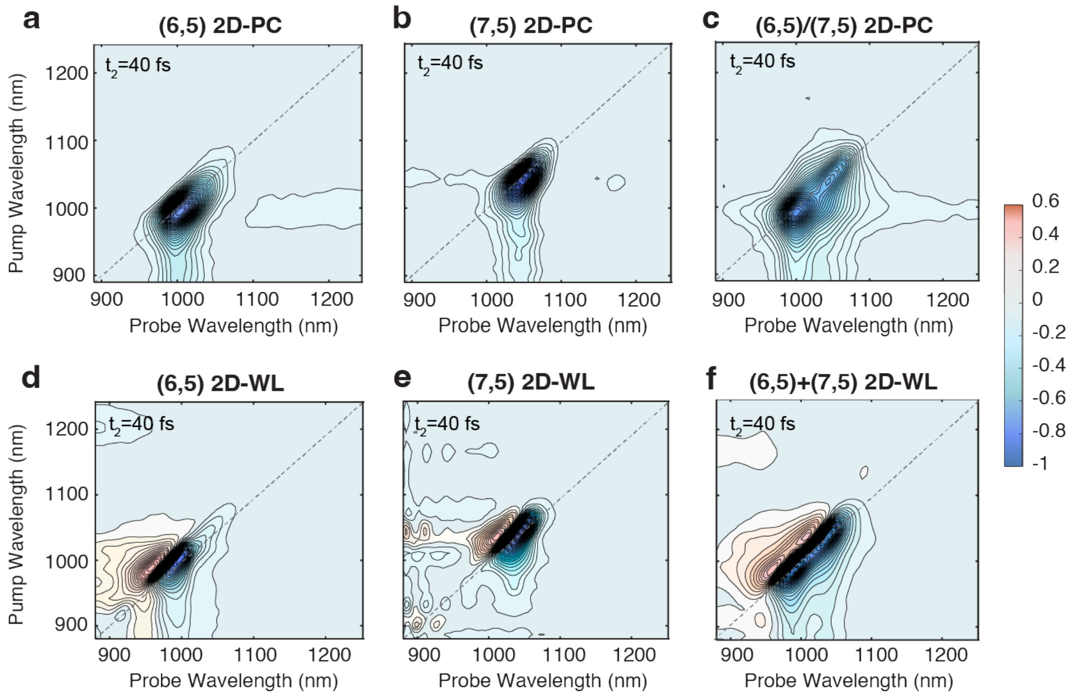

Supplementary Fig. 6: Depicts pairs of normalized 2D photocurrent and 2D white light spectra. The 2D photocurrent and 2D white light spectra in each pair were collected simultaneously. Each spectra presented in this figure was collected at a t_2 delay of 40fs. (a,d) were collected using the (6,5) single chirality device. (b,e) were collected using the (7,5) single chirality device. (c,f) were collected using the (6,5)/(7,5) mixed chirality device.

Supplementary Figure 7: Hole Dynamics at long t_2 Delays

Shown in Supplementary Fig. 7 below are transient photocurrent and 2D photocurrent kinetics of the mixed (6,5)/(7,5) device. The left column of kinetics data (Supplementary Fig. 7a,d) is transient photocurrent data, the middle column of kinetics data (Supplementary Fig. 7b,e) is 2D photocurrent of diagonal peaks, and the right column of kinetics data (Supplementary Fig. 7c,f) is 2D photocurrent of upper and lower cross peaks. Supplementary Fig. 7a-c depicts the full 100ps kinetics while Supplementary Fig. 5d-f shows only the data at t2 delays that are greater than 1ps. Two 2D photocurrent spectra (Supplementary Fig. 7g,h) are also shown as representative spectra at long t_2 delays.

In this mixed (6,5)/(7,5) device, we observe negative features appear at long t_2 delays that do not appear in single chirality devices. For example, the transient photocurrent kinetics become negative at ∼8ps and continue to decrease before beginning to plateau at later t_2 delays while the single chirality data above shows no sign of dropping below zero. Transient photocurrent spectroscopy does not have the ability to resolve diagonal peaks from cross peaks under this polarization scheme. To do so, we turn to 2D photocurrent spectroscopy. Upon doing so, we find that the diagonal peaks never become negative, and their kinetics appear similar to the single chirality transient data reported. On the other hand, the upper and lower cross peak in the 2D photocurrent data do become negative, which can be observed in both the kinetics traces and the 2D spectra. Taken at face value, these types of features suggest more current is being generated at long time delays after the pump. One mechanism through which this could take place is trap state filling. If carriers generated from the pump fill trap states over the course of tens of picoseconds, then the probe has the potential to generate more current. This mechanism, however, is only a hypothesis and will need to be tested in future works.

In both the transient and 2D photocurrent kinetics, we also observe that at long t_2 delays the (6,5) features begin decaying faster than the (7,5) features. First, in the transient photocurrent data, this trend can be seen as the (6,5) kinetics start becoming less negative than the (7,5) kinetics. Next, in the 2D photocurrent data, the (6,5) diagonal peak begins decaying faster than the (7,5) peak at long t_2 delays. Finally, in the 2D photocurrent data, the upper cross peak decays faster than the lower cross peak after they both become negative. This difference in cross peak decay rates also means that at long t_2 delays, they are no longer symmetric like was observed in Fig. 3 in the main text. This observation is made very clear by examining the mixed (6,5)/(7,5) 2D photocurrent spectra at long t_2 delays. The differences in decay rates observed here could be caused by preferential hole filling in (6,5) CNTs from the anode or inhomogeneity in the mixed CNT layer that preferentially places (6,5) CNTs closer to the anode on average.

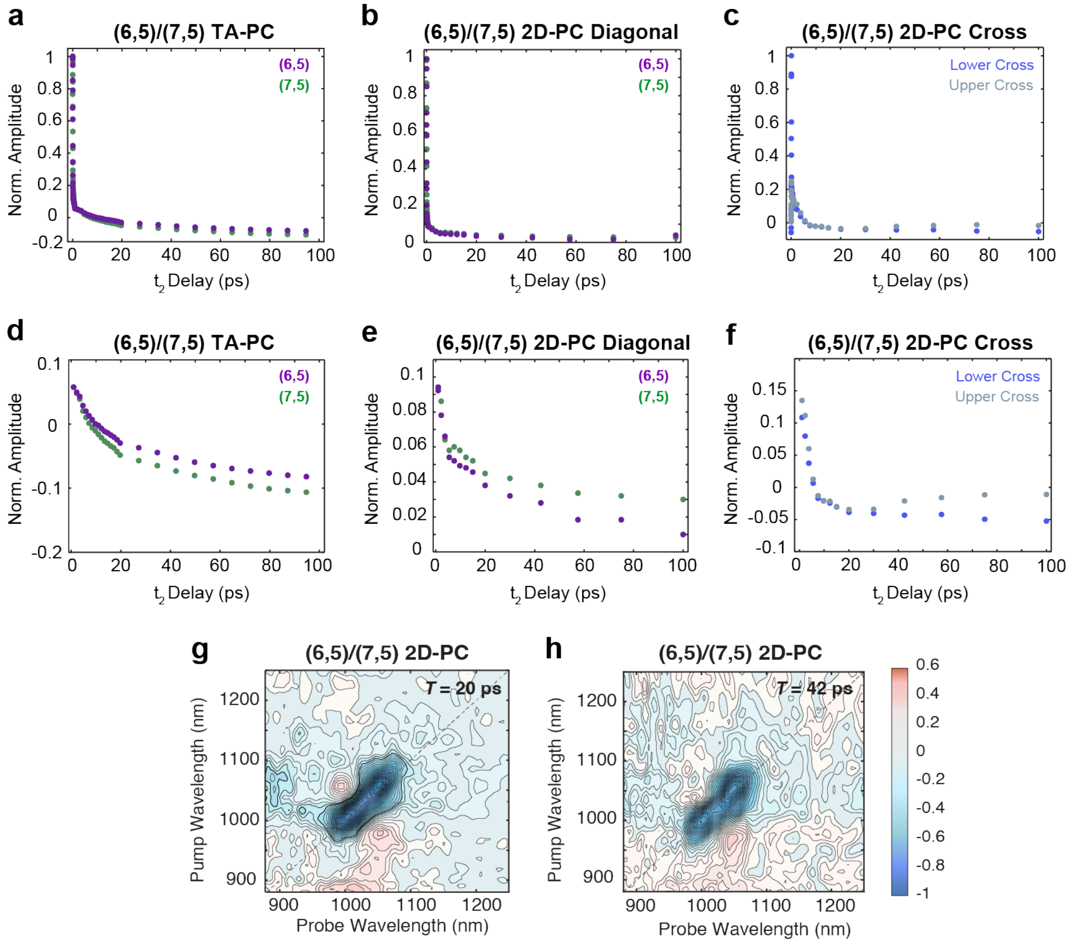

Supplementary Fig. 7: Depicts the transient photocurrent and 2D photocurrent kinetics of the (6,5)/(7,5) mixed chirality device as well as normalized 2D photocurrent spectra taken at long t_2 delays. (a,c) contain full 100ps photocurrent data while (d,f) contain photocurrent data take from t_2 delays greater than 1 ps. (g,h) are normalized 2D photocurrent spectra taken at t_2 delays of 20 and 42ps.

131 # Supplementary Figure 8: Optical Apparatus

132 The figure below depicts the ultrafast spectrometer described in the Methods section of the main text.

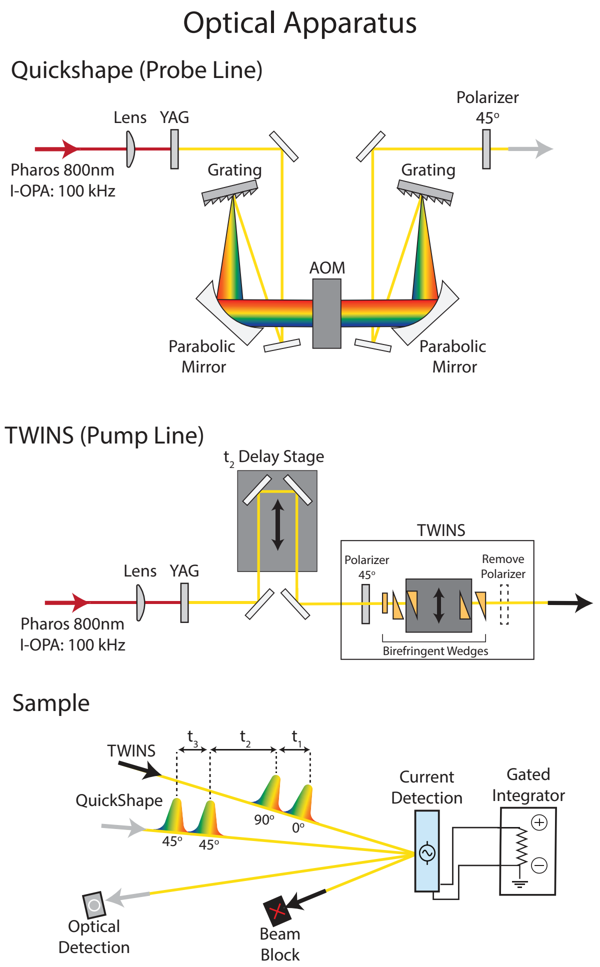

Supplementary Fig. 8: Depicts the optical apparatus used to perform ultrafast photoabsorption and photocurrent measurements. The diagram is split into three sections: Quickshape (Probe Line), TWINS (Pump Line), and Sample. The Quickshape (Probe Line) section depicts a transverse AOM-based pulse shaper set to 45° polarization. The TWINS (Pump Line) section depicts t_2 delay stage as well as the TWINS birefringent interferometer with the second polarizer removed. The Sample section depicts the final pulse sequence and the two modes of detection (photoabsorption and photocurrent).

Additional Equations

Kinetics Model

Below are the integrated rate equations used to fit the kinetics data.

$$P_{\text{recom}}(t) = Ae^{-(\frac{t}{a})^{1/2}} \tag{1}$$

$$P_{\text{dis}}(t) = Be^{-bt} + Ce^{-ct} \tag{2}$$

$$H(t) = -P_{\text{dis}} + De^{-dt} + Ee^{-et} \tag{3}$$

where A-E are the relative amplitudes of the exponentials. These variables will change depending on the measurement (optical film, optical device, photocurrent device) being fit. As an additional note, ∼2% of excitons were found to spontaneously dissociate in the thin film.[55] These excitons were accounted for with an offset in the thin film fit but do not appear in the kinetic equations. The fits for the kinetics data using these exponentials are calculated using the equation below.

$$\text{Film Abs.} = P_{\text{recom}}(t) \tag{4}$$

$$\text{Device Abs.} = P_{\text{recom}}(t) + P_{\text{dis}}(t) + \frac{1}{2}H(t) \tag{5}$$

$$\text{Device Current} = P_{\text{dis}}(t) + \frac{1}{2}H(t) \tag{6}$$

In the film absorption fit, A=1, in the device absorption fit, $A + B + C = 1$ and $D + E = 1$, and in the device current fit, $B + C = 1$ and $D + E = 1$. The factor of $\frac{1}{2}$ appears in front of$H(t)$ because holes cause half the bleach compared to excitons as described in Supplementary Fig. 3 above.

Calculating Errors in Fits

In this work, we calculate the standard error (SE) of each fit parameter using the following formula.

$$\text{SE} = \pm\sqrt{\frac{SS}{DF} * \text{COV}(i,i)}$$

where SS is the sum of square residuals, DF is the degrees of freedom (number of data points minus number of variable parameters), and COV(i,i) are the diagonal elements of the covariance matrix. These diagonal elements represent the variance of each parameter used in the fit. The covariance matrix can be calculated from the following formula.

$$\text{COV} = H^{-1} = (AA^T)^{-1}$$

where H is the Hessian and A is the design matrix. The elements of the design matrix can be calculate using the formula below.

$$A_{ij} = \frac{\frac{\partial Y}{\partial P_i}}{\sigma(y_j)}$$

where y is the fit function, P_i is the ith parameter in the fit function, and $\sigma(y_j)$ is the variance of the jth data point being fit. The standard errors reported in this work are rounded to the most significant digit.

Calculating Exciton Density after the Pump Pulse

We calculate the number of CNTs/cm^2 for a 4nm film based on the density of the film (2g/cm^3). Based on these metrics, a 1cm^2 film is 0.8μg. The films are 40% CNT, so a given film is comprised of 0.32μg of carbon (or 26.67 nmol of carbon). 26.7 nmol of carbon is $1.6 * 10^{16}$ carbon atoms. The average length of the (6,5) CNTs used in this study is 500nm, which each contain $5 * 10^4$ carbon atoms based on the number of CNT unit cells in an average length CNT. With these numbers in hand, we calculate $3.2 * 10^{11}$ CNTs/cm^2 in our 4nm thick films. Our absorbed pump fluence is $4 * 10^{11}$, so there are on average 1.25 excitons/CNT, or one exciton every 400 nm.
